# Dynamic Changes in Ascorbic Acid Content during Fruit Development and Ripening of *Actinidia latifolia* (an Ascorbate-Rich Fruit Crop) and the Associated Molecular Mechanisms

**DOI:** 10.3390/ijms23105808

**Published:** 2022-05-22

**Authors:** Honghong Deng, Hui Xia, Yuqi Guo, Xinling Liu, Lijin Lin, Jin Wang, Kunfu Xu, Xiulan Lv, Rongping Hu, Dong Liang

**Affiliations:** Institute of Pomology and Olericulture, College of Horticulture, Sichuan Agricultural University, Chengdu 611130, China; denghh@sicau.edu.cn (H.D.); xiahui@sicau.edu.cn (H.X.); 2020305047@stu.sicau.edu.cn (Y.G.); 2020205019@stu.sicau.edu.cn (X.L.); llj800924@sicau.edu.cn (L.L.); 14224@sicau.edu.cn (J.W.); 71389@sicau.edu.cn (K.X.); 10998@sicau.edu.cn (X.L.); 2020305047@stu.edu.cn (R.H.)

**Keywords:** *Actinidia latifolia*, HPLC, ascorbic acid accumulation, PacBio SMRT sequencing, full-length transcriptome, ascorbic acid biosynthesis, recycling

## Abstract

*Actinidia latifolia* is one of the very few kiwifruit genotypes with extremely high ascorbic acid (AsA) content. However, a transcriptome atlas of this species is lacking. The accumulation of AsA during fruit development and ripening and the associated molecular mechanisms are still poorly understood. Herein, dynamic changes in AsA content at six different stages of *A. latifolia* fruit development and ripening were determined. AsA content of *A. latifolia* fruit reached 1108.76 ± 35.26 mg 100 g^−1^ FW at full maturity. A high-quality, full-length (FL) transcriptome of *A. latifolia* was successfully constructed for the first time using third-generation sequencing technology. The transcriptome comprises 326,926 FL non-chimeric reads, 15,505 coding sequences, 2882 transcription factors, 18,797 simple sequence repeats, 3328 long noncoding RNAs, and 231 alternative splicing events. The genes involved in AsA biosynthesis and recycling pathways were identified and compared with those in different kiwifruit genotypes. The correlation between the AsA content and expression levels of key genes in AsA biosynthesis and recycling pathways was revealed. LncRNAs that participate in AsA-related gene expression regulation were also identified. Gene expression patterns in AsA biosynthesis and metabolism exhibited a trend similar to that of AsA accumulation. Overall, this study paves the way for genetic engineering to develop kiwifruits with super-high AsA content.

## 1. Introduction

L-ascorbic acid (AsA, L-threo-hex-2-enono-1,4-lactone), also known as vitamin C and ascorbate, is an essential water-soluble, low molecular weight antioxidant [1] which supports plant growth and development [2] and the response of plants to environmental stresses [3]. In addition, AsA prevents and alleviates various human ailments and diseases, including some chronic and complex diseases (stroke, cancer, and scurvy) [4], and even has possible physiological effects on severe coronavirus disease 2019 (COVID-19) [5]. Throughout evolution, humans and other primates have lost the ability to synthesize AsA by themselves due to a lack of the L-gulonolactone oxidase (*GLO*) gene, and are thus entirely dependent upon food supplies, such as plant products [6]. Horticulture plants, particularly fresh fruits and vegetables, are abundant sources of AsA for human beings, sufficiently satisfying daily AsA requirements [7]. The benefits of the increase in AsA production in plants are profound.

Kiwifruit is a member of the genus *Actinidia* Lindl. belonging to the family Actinidiaceae. It is an important fruit crop widely distributed throughout most of East Asia, with the center of evolution being the Yangzi and Pearl Rivers and mountain ranges in China [8]. Kiwifruit has long been considered “the king of fruits” because of its remarkably high AsA content [8], and the presence of other nutritional phytochemicals that can provide several health benefits [9,10]. When compared with other commercially important fruit crops, the AsA content in equal fresh weight (FW) kiwifruit is approximately one-to-two times higher than that of orange, while it is 10 times higher than that of banana and up to 15 times higher than that of apple [11,12]. Within the genus *Actinidia* Lindl., there is great natural variation in AsA content. However, *A. latifolia* is one of the very few kiwifruit genotypes that are regarded as an exceptional source of extremely high AsA [13,14,15,16]. Accordingly, *A. latifolia* could serve as a valuable source for improving AsA content in other commercially important kiwifruit cultivars in future breeding strategies.

**Figure 1:**
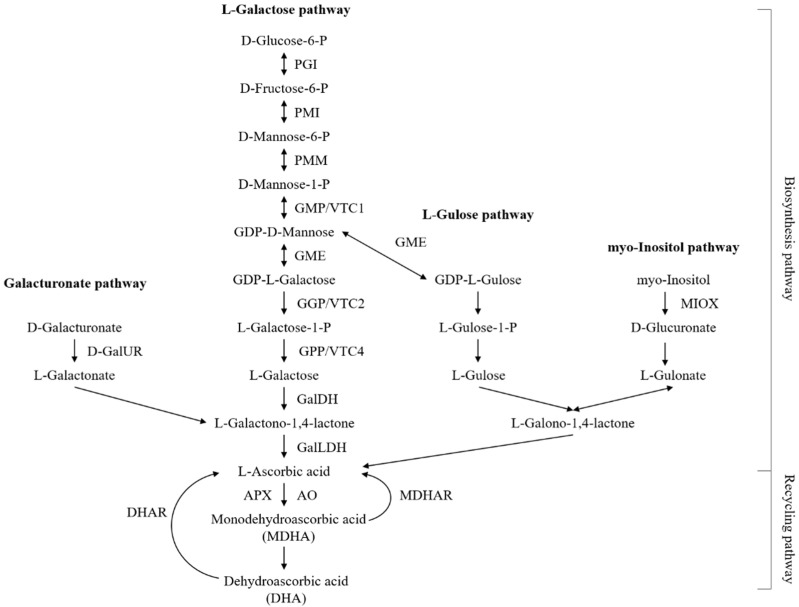
The currently known pathways for AsA biosynthesis and recycling in higher plants. Abbreviations: Phosphoglucoisomerase, PGI; mannose-6-phosphate isomerase, PMI; phosphomannomutase, PMM; GDP-mannose pryophosphorylase, GMP; GDP-mannose-3′,5′-epimerase, GME; GDP-L-galactose phosphorylase, GGP; L-galactose-l-phosphate phosphatase, GPP; L-galactose dehydrogenase, GalDH; L-galactono-1,4-lactone dehydrogenase, GalLDH; myo-inositol oxygenase, MIOX; D-galacturonate reductase, GalUR; ascorbate peroxidase, APX; monodehydroascorbate reductase, MDHAR; dehydroascorbate reductase, DHAR; ascorbate oxidase, AO.

Several transgenic approaches have been employed to determine the molecular mechanisms underlying the AsA biosynthesis pathways in higher plants. The current consensus is that four AsA biosynthesis pathways, including the L-galactose, L-gulose, D-galacturonic acid, and inositol pathways, operate in higher plants, with the L-galactose pathway being the dominant route for AsA accumulation [1], as illustrated in Figure 1.

In recent decades, de novo assembly of the transcriptome by second-generation sequencing (NGS), Illumina RNA-sequencing (RNA-Seq), has become a cost-effective, accurate, and routine method. Nevertheless, NGS generates relatively short reads of only 100–500 bp, which poses a great challenge when it comes to assembly and annotation, especially without a well-annotated reference genome [17]. Recently, the advent of a third-generation sequence platform, PacBio long-read single-molecule real-time (SMRT) sequencing technology, has overcome the limitations of short read sequences and provides opportunities to generate reliable long reads (10–15 kb) which can meet the requirements for capturing genome-wide, full-length (FL) transcripts [17]. Although *A. latifolia* has extremely high AsA content [13,14,15,16], neither its reference genome nor transcriptome is available, which is a major bottleneck in understanding the functional genomics and molecular genetics of *A. latifolia*. To the best of our knowledge, there are no reports regarding the AsA accumulation pattern in this special AsA-rich kiwifruit cultivar. To elucidate the mechanisms regulating AsA concentration, it is of utmost importance to monitor how AsA accumulates during fruit development and ripening of cultivars with different AsA contents, especially in cultivars with extremely high AsA content. In addition, much remains to be learned regarding the molecular mechanisms underlying the dynamic accumulation of AsA in *A. latifolia*. A thorough elucidation of AsA accumulation, its associated gene expression, and metabolic pathways is required for genetic or cultural improvement in the future.

In the present study, the dynamic changes in AsA content at six different stages of fruit development and ripening of *A. latifolia* were evaluated using high-performance liquid chromatography (HPLC). The Isoform Sequencing (Iso-Seq^TM^) protocol with the PacBio SMRT sequencing-based de novo transcriptome was employed to construct a high-quality reference FL transcriptome for *A. latifolia*. In addition, the associated transcriptional changes, along with AsA accumulation, were quantified using second-generation Illumina paired-end sequencing technology to elucidate the underlying molecular mechanisms. Furthermore, the regulatory genes associated with the AsA biosynthesis and recycling pathways, and the lncRNAs involved in AsA-related gene expression regulation, were identified. The paucity of *A. latifolia* genomic information implies that the reference FL transcriptome atlas obtained in this study would be vital for future genome annotation and studying gene function, especially those related to economically important traits. The results of this study would be beneficial for breeding programs aimed at increasing the AsA level of kiwifruit in the future.

## 2. Results

### 2.1. Morphological Changes and AsA Accumulation at Six Different Stages of the Fruit Development and Ripening of A. latifolia

**Figure 2:**
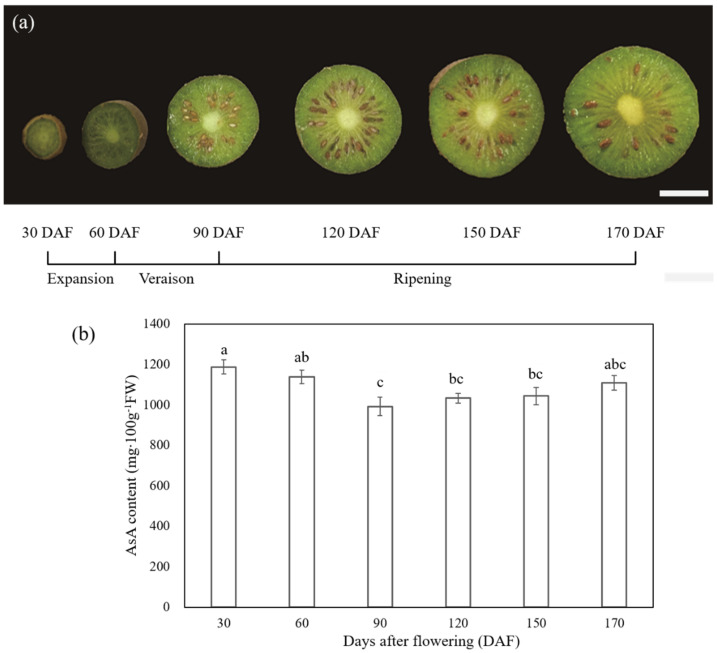
Phenotypic characterization (**a**) and AsA accumulation (**b**) of *A. latifolia* fruit development and ripening. The x-axis represents different stages of fruit development and ripening, which are expressed as days after flowering. The scale bar in (**a**) denotes 1 cm. Vertical error bars in (**b**) represent standard error (SE) of three biological replicates. The significance of differences (*p* < 0.05) among samples were determined using ANOVA and Tukey’s HSD test. Different letters on the vertical bars indicate significant difference.

Visual inspection of six different stages corresponding to 30, 60, 90, 120, 150, and 170 DAF of *A. latifolia* fruit development and ripening is shown in Figure 2a. During development, *A. latifolia* fruit underwent three distinguishable phases, including a cell division and cell expansion phase from fruit set to approximately 60 DAF; a veraison phase with fruit color changing (60–90 DAF); and a ripening phase (90–170 DAF), as displayed in Figure 2a. AsA content was quantified by HPLC (Figure 2b). A significant (*p* < 0.05) decrease in AsA content occurred during the expansion and veraison stages, reaching the lowest level at 90 DAF (992.08 ± 46.59 mg 100 g^−1^ FW). As ripening progresses and the fruits transition to maturation, the AsA content showed an increasing trend from 90 to 170 DAF, and reached 1108.76 ± 35.26 mg 100 g^−1^ FW at full maturity. However, the statistical evaluation results of ANOVA showed no significant differences. The details of the changes in AsA content over time are expressed as the mean ± standard error (SE) on a FW basis, and are shown in Figure 2b.

### 2.2. PacBio SMRT Sequencing-Based FL Transcriptome Atlas of A. latifolia Fruit

**Figure 3:**
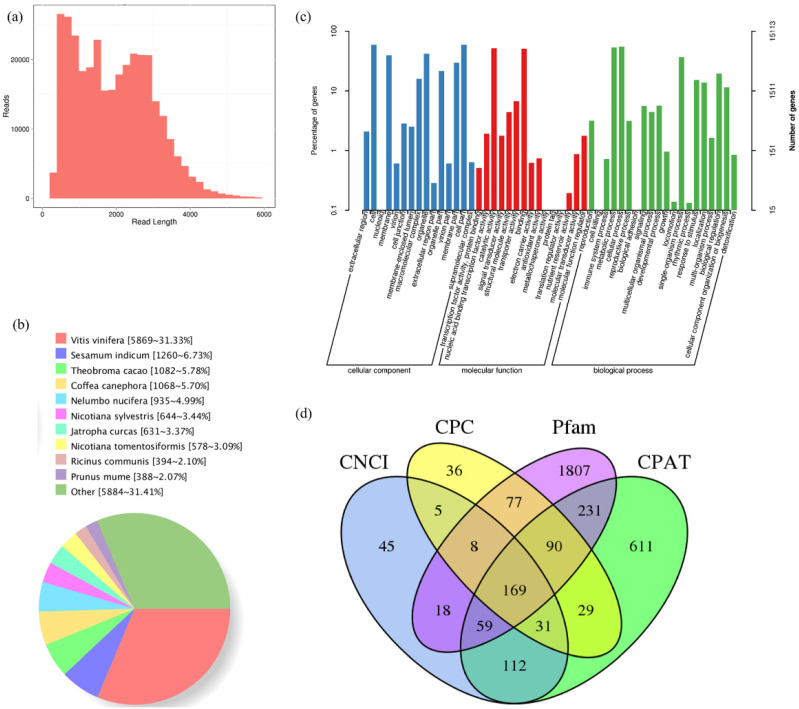
Overview of characteristics of *A. latifolia* fruit FL transcriptome. (**a**) Consensus isoform read-length distribution of 1–6 K size bin. (**b**) Homologous species distribution of *A. latifolia* transcripts annotated in the non-redundant database. (**c**) The alignment results against the gene ontology (GO) database. (**d**) Candidate lncRNAs predicted by CNCI, CPC, Pfam, and CPAT database.

To capture as many transcripts of *A. latifolia* fruit as possible, a total of eighteen RNA samples from six representative stages of fruit development and ripening of *A. latifolia* were equally pooled for library preparation (1–6 kb libraries). A total of 19.50 Gb of subreads were obtained from the offline data. The strict screening criteria (i.e., full passes greater than 1.0 and accuracy greater than 0.90) resulted in 358,138 CCS reads, comprising 743,399,287 read bases, with an average read length of 2087 bp. After error correction, a total of 34,148 polished high-quality isoforms were obtained, among which 91.80% (326,926 reads) were filtered as the FLNC reads of *A. latifolia* fruit transcriptome. The FLNC read-length distribution ranged from 700 to 3500 bp, and the overall distribution of each bin size agreed with the size of its cDNA library (Figure 3a).

### 2.3. Functional Annotation of A. latifolia Transcripts with Multiple Databases

Transcripts were scanned against and successfully annotated using the Nr, GO, COG, KOG, eggNOG, KEGG, Pfam, and Swiss-Prot databases, and the integrated alignment results are summarized in Table S2. Based on Nr functional annotation, the best BLAST hit of homologous species with *A. latifolia* fruit was *Vitis vinifera* (5689 isoforms, 31.33%), followed by *Sesamum indicum* (1260, 6.73%), and *Theobroma cacao* (1082, 5.78%) (Figure 3b). In total, 15,113 transcripts were successfully annotated using the GO database and classified into three categories: biological process (BP), cellular component (CC), and molecular function (MF). Genes involved in cellular process (8291 matched genes, 54.86%), metabolic processes (8072, 53.41%), and single-organism processes (5540, 36.66%) were highly represented in the BP category. Cells (8924 matched genes, 59.05%) were the most abundant subcategory within CC, followed by cell parts (8911, 58.96%) and organelles (6340, 41.95%). In the MF category, catalytic activity (7823 matched genes, 51.76%) was the most prominent category, followed by binding (7672, 50.76%), and transporter activity (1023, 6.77%) (Figure 3c).

### 2.4. Structural Analysis of the FL Transcriptome of A. latifolia

A total of 15,505 CDS were identified. The frequencies for each CDS length were evaluated, with the most frequent length ranging from 100 to 1200 bp (Figure S1a). Furthermore, by predicting non-redundant transcripts using the iTAK software, 2436 genes were predicted to be TFs. These TFs were classified into different families, among which the most abundant type identified was C3H (84 matched genes), followed by GRAS (76), AP2/ERF-ERF (68), MYB-related (56), B3-ARF (56), CAMK_CDPK (55), C2H2 (54), and RLK-Pelle_LRR_Xl-1 (51) (Figure S1b). A total of 3328 lncRNA candidates were identified, and 447, 445, 2459, and 1332 lncRNAs were identified using CNCI, CPC, Pfam, and CPAT prediction results, respectively. The comparison revealed that 169 transcripts were simultaneously identified using the four computational approaches (Figure 3d). In addition, transcripts were subjected to SSR analysis via MISA, and a total of 18,797 SSR, including seven SSR types (i.e., mononucleotide, dinucleotide, trinucleotide, tetranucleotide, pentanucleotide, hexanucleotide, and compound nucleotides) were detected. The dinucleotide SSR loci exhibited the highest frequency, followed by the mononucleotide and the compound SSR types (Figure S1c). A detailed breakdown of the SSR types from PacBio is presented in Table S3.

### 2.5. Differentially Expressed Genes (DEGs) Identified in Comparative Transcriptomic Analysis

The non-redundant transcripts obtained were used as a reference for Illumina sequence alignment and subsequent analysis. Eighteen cDNA libraries for Illumina sequencing were generated from six different stages, with three biological replicates per stage. DEG identification was independently performed using pair-wise comparisons between the developmental stage and baseline control. Based on the adopted cutoff (FDR < 0.01 and absolute fold change ≥ 2), the numbers of all genes displaying both significantly up- and down-regulated profiles are summarized in Table 1.

### 2.6. Functional Annotation and Categorization of DEGs

The annotation, pathway, and functional categorization of the DEGs in the comparisons of DAF30 versus DAF60, 90, 120, 150, and 170 were thoroughly analyzed using the COG, GO, KEGG, KOG, Pfam, Swiss-Prot, eggNOG, and Nr databases. The assignments indicated that at least 97.79% of the DEGs were functionally annotated in multiple databases (Table 2). Analysis of GO categories showed that the functional distribution of DEGs in comparable groups was similar. The BP categories of GO terms were primarily grouped into cellular, metabolic, and single-organism processes. The CC category was mainly assigned to cells, cell parts, and membranes. Catalytic, binding, and transporter activities were prominent in the MF category (Figure S2). KEGG pathway enrichment analysis, a pathway-based categorization of orthologous genes, was conducted to predict the functional profiles and biological significances of DEGs identified during the six different stages of *A. latifolia* fruit development and ripening. Plant hormone signal transduction, carbon metabolism, amino acid biosynthesis, starch and sucrose metabolism, and protein processing in the endoplasmic reticulum were the most significantly enriched metabolic pathways (Figure S3).

### 2.7. Identification of Genes in AsA Biosynthesis and Recycling Pathway during the Fruit Development and Ripening of A. latifolia

By combining second- and third-generation sequencing technologies, crucial regulatory genes associated with AsA biosynthesis and recycling pathways were identified. All genes in the L-galactose pathway, some key genes in the other three pathways, and genes in the AsA recycling pathway were successfully identified during fruit development and ripening of *A. latifolia*. The number of these gene families in different kiwifruit genotypes, including Hongyang (*A. chinensis*) v2, Hongyang (*A. chinensis*) v3, Red 5 (*A. chinensis*), White (*A. eriantha*), and *A. latifolia*, were compared, and the results are listed in Table 3. At least two gene family members in *A. latifolia* were identified, except for GalDH and GalLDH. Gene expansions were detected not only in PMM, GMP, and GGP within the L-galactose pathway, which forms the major route for ascorbic acid biosynthesis, but also in genes involved in the recycling of ascorbic acid, including AO, APX, and MDHAR (Table 3).

**Figure 4:**
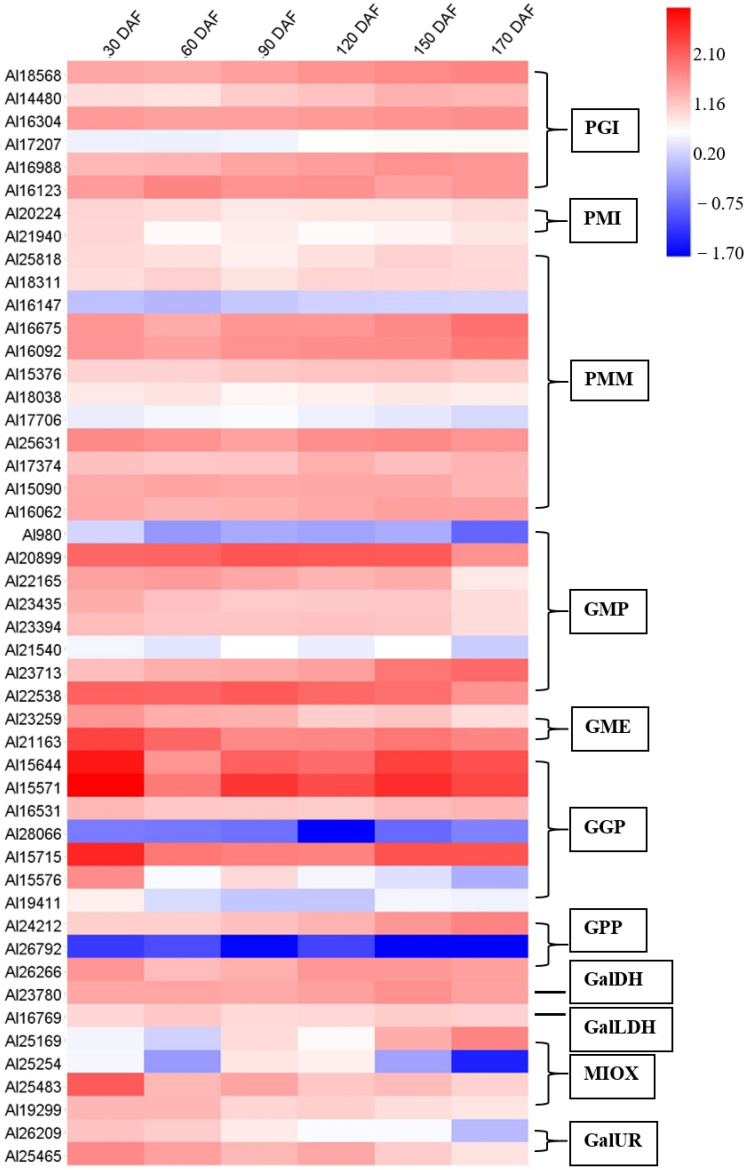
Heat map depicting the expression profiles of AsA biosynthesis-related genes during fruit development and ripening of *A. latifolia*. The mean values from transcriptome data of the triplicates were used for heat map construction. The expression level was represented by a color scale located at the top right corner.

**Figure 5:**
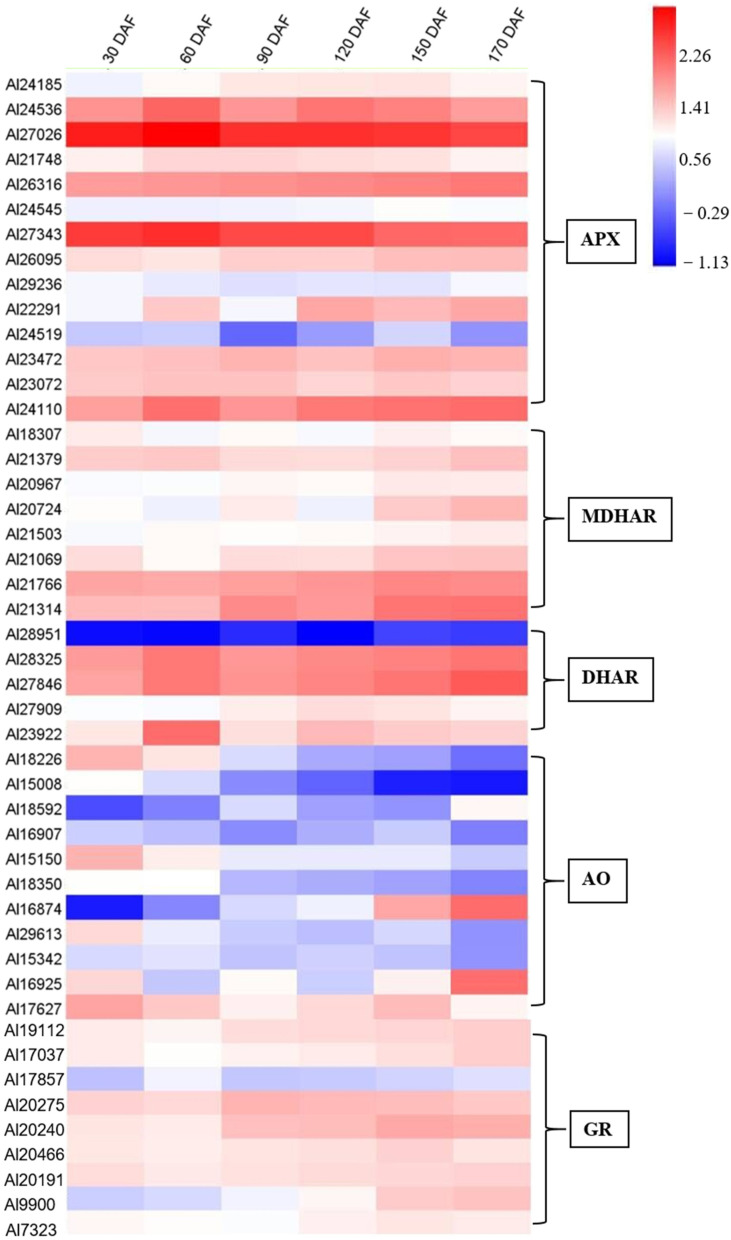
Heat map depicting the expression profiles of AsA recycling-related genes during fruit development and ripening of *A. latifolia*. The mean values from transcriptome data of the triplicates were used for heat map construction. The expression level was represented by a color scale located at the top right corner.

Changes in the relative expression of gene family members involved in AsA biosynthesis and recycling over time were investigated in this study and their expression profiles are summarized in the heat maps (Figure 4 and Figure 5), except for PMM (Al31169), GME (Al24721), GGP (Al33117), APX (Al27791), APX (Al28761), APX (Al30425), MDHAR (Al29807), and MDHAR (Al22533). In the AsA biosynthesis pathway, GMP (Al20899, Al23713, and Al22538), GME (Al21163), and GGP (Al15644, Al15571, and Al15715) were highly expressed during fruit development and ripening of *A. latifolia* (Figure 4). In the AsA recycling pathway, APX (Al24536, Al27026, Al26316, Al27343, and Al24110), MDHAR (Al21766 and Al21314), and DHAR (Al28325 and Al27846) showed high transcriptional levels (Figure 5).

The statistical correlation or association between AsA accumulation and relative gene expression was measured and is summarized in Table S4. Positive highly significant (*p* < 0.05) correlations between the AsA content and gene expression were observed in two *PMI* genes, i.e., Al20224 [correlation coefficient (r) = 0.861] and Al21940 (r) = 0.523, two *PMM* genes, [Al18038 (r) = 0.601 and Al25631 (r) = 0.496], one *GMP* gene, [Al23435 (r) = 0.559], two *GME* genes, [Al23259 (r) = 0.507 and Al21163 (r) = 0.722], five *GGP* genes, [Al15644 (r) = 0.505, Al16531 (r) = 0.567, Al15715 (r) = 0.632, Al15576 (r) = 0.528, and Al19411 (r) = 0.625], two *MIOX* genes, [Al19299 (r) = 0.669 and Al25483 (r) = 0.580], two *GalUR* genes, [Al26209 (r) = 0.574 and Al25465 = 0.595], two *APX* genes, [Al27026 (r) = 0.470 and Al29236 (r) = 0.56), one *MDHAR* gene, [Al21379 (r) = 0.635], and six *AO* genes, [Al18226 (r) = 0.713, Al15008 (r) = 0.691, Al15150 (r) = 0.676, Al18350 (r) = 0.694, Al29613 (r) = 0.630 and Al17627 (r) = 0.580]. However, the expression levels of one *PGI* gene, [Al16988 (r) = −0.517], one *PMI* gene, [Al15376 (r) = −0.651], two *GMP* genes, [Al20899 (r) = −0.508 and Al21540 (r) = −470], one *APX* gene, [Al24185 (r) = −0.765], and one *DHAR* gene, [Al27909 (r) = −0.618], showed significant (*p* < 0.05) negative correlations. The correlation analysis results revealed that three optional biosynthesis and recycling pathways (L-galactose, galacturonate, and myo-inositol pathways) were significantly associated with AsA accumulation during fruit development and ripening of *A. latifolia*.

### 2.8. LncRNAs Participated in AsA-Related Gene Expression Regulation

There were 169 lncRNA candidates identified by the intersection of the CNCI, CPC, Pfam, and CPAT prediction results (Figure 3d), 91 of which may act on downstream target genes. In all downstream target genes, we found many genes involved in the AsA L-galactose pathway and recycling, such as PGI, PMM, GMP, GME, GGP, GPP, APX, MDHAR, DHAR, and AO (Table S5). Several lncRNAs can act on multiple target genes. For example, Al32641 and Al32633 have six target genes. In addition, many functional genes can be regulated by multiple lncRNAs, such as Al16988 (PGI), Al15576 (GGP), and Al15150 (AO). These results indicate that lncRNAs play important roles in AsA accumulation during fruit development and ripening of *A. latifolia* via complex regulatory networks.

### 2.9. Expression Patterns of Genes Involved in AsA Biosynthesis and Recycling during Fruit Development and Ripening of A. latifolia Verified by qRT-PCR Analysis

**Figure 6:**
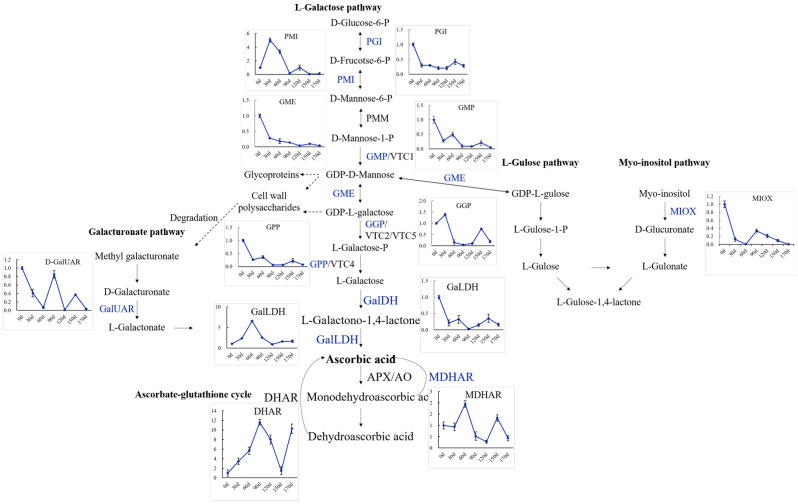
qRT-PCR validation of the expression profiles of candidate DEGs associated with AsA biosynthesis and metabolism in *A. latifolia*. Gene abbreviations are the same as those in Figure 1.

To investigate the molecular mechanisms regulating AsA biosynthesis during the fruit development and ripening of *A. latifolia*, the expression levels of AsA biosynthesis genes at different developmental stages were further examined by qRT-PCR, as illustrated in Figure 6. The L-galactose pathway, L-gulose, myo-inositol, and D-galacturonic acid pathways were identified. The overall gene expression levels of eight enzymes, including *GalDH*, *GGP*, *GME*, *GPP*, *GMP*, *PGI*, *MIOX*, and *APX* (Figure 6), exhibited a similar trend to the cumulative AsA content described above (Figure 2b). The L-galactose and myo-inositol pathways were the predominant pathways regulating the high AsA content in *A. latifolia* fruit development and ripening (Figure 6).

## 3. Discussion

### 3.1. The First High-Quality, Functionally Annotated Reference Transcriptome for A. latifolia

*A. latifolia* has been identified as a promising kiwifruit species with remarkably high AsA concentration [13,14,15,16]. The lack of a high-confidence transcriptome atlas of *A. latifolia* greatly hindered scope of investigation into the molecular genetic basis of this important cultivar. In this study, we successfully built a high-quality transcriptome for *A. latifolia* for the first time using PacBio SMRT sequencing technology corrected by RNA-seq (Figure 3), which will be a crucial resource in exploring genome mining and understanding gene functions for this species.

The reference transcriptome and draft genome data of *A. latifolia* have not yet been completely sequenced. It is crucial to annotate the transcripts for biological functions and metabolic pathways. Therefore, sequence-based alignments were conducted against eight functional databases (Figure 3) to understand the high-level functions and utilities of biological systems. Another important aspect of our study was the prediction of CDS, TF, and lncRNA from the non-redundant transcripts of *A. latifolia* (Figure S1). LncRNAs represent a novel class of non-coding RNA that regulate a range of biological processes, such as plant growth, development, and stress responses [18]. In the present study, lncRNAs were identified using a combination of the CPC, CNCI, Pfam, and CPAT databases (Figure 3d), which will be useful for subsequent studies on the biological functions of lncRNA in *A. latifolia*.

The dinucleotide SSR type was the most frequently observed type in *A. latifolia*, which was in agreement with the expressed sequence tag (EST)-derived SSR distribution previously reported in *Actinidia* species [19]. SSR markers have proven to be the most favored molecular markers for estimating genetic diversity, phylogenetic relationships, genotype identification and discrimination, marker-phenotype association, and genetic map construction [20]. The FL transcriptome atlas contains a large amount of genetic information and is a rich source of SSR discovery [21]. Transcriptome-based SSR development has increased the potential for association with functional genes or even agronomic phenotypes because of the close linkage to expressed genes in the transcriptome [22]. This study reported the use of PacBio SMRT sequencing technology for discovery of a set of SSR loci in *A. latifolia* for the first time (Table S3). The SSR markers identified in this study will be a valuable resource for marker-assisted breeding of *A. latifolia*.

### 3.2. Dynamics of AsA Content Accompanying Fruit Development and Ripening of A. latifolia

AsA plays a plethora of roles in biological functions of both plants and humans [2,3,4,5]. Besides cultivar-dependent differences [13,14,15,16], kiwifruits also show tissue- and developmental-specific variability in AsA content [23]. The highest accumulation of AsA was recorded at four weeks after anthesis in *A. chinenesis* (50–200 mg 100 g^−1^ FW) and at six weeks after anthesis in *A. eriantha* (800 mg 100 g^−1^ FW) and *A. deliciosa* (80 mg 100 g^−1^ FW) [23]. *A. chinensis* var. *deliciosa* ‘Qinmei’ synthesizes AsA primarily during the early fruit development stage [24]. Zhang et al. [25] reported that *A. chinensis* var. *chinensis* ‘Hongyang’ kiwifruit exhibited a maximal AsA level at its immature green stage due to the high biosynthesis rate, which decreased as it ripened and then remained fairly stable until complete ripening.

In the present study, we quantified the dynamic changes in AsA by HPLC during different stages of fruit development and ripening of *A. latifolia* (Figure 2). Although no significant changes in AsA content were detected by Tukey’s HSD test during the fruit ripening stage (90–170 DAF), there was a rapid decreasing profile during the initial expansion (30–60 DAF) and veraison stage (60–90 DAF), followed by a progressive, albeit not significant, increasing tendency (Figure 2b). Overall, the accumulation dynamic of AsA in *A. latifolia* positively correlated with fruit developmental stages, as in other kiwifruit varieties [23,24,25]. On the other hand, our results demonstrated the extremely high levels of AsA content of *A. latifolia* (1108.76 ± 35.26 mg 100 g^−1^ FW, Figure 2b), which were consistent with previous studies [13,14,15,16].

### 3.3. An Elucidation of the Molecular Mechanisms Regulating AsA Accumulation of A. latifolia

To date, much progress has been made toward understanding AsA biosynthesis and recycling in higher plants [26]. At least four distinct metabolic pathways, including the L-galactose, L-gulose, galacturonic, and myo-inositol pathways, form a complex network for AsA biosynthesis [1]. AsA biosynthesis and metabolism are complex reactions that depend on the co-expression and coordination of a cluster of genes [1,4,12]. All the genes encoding enzymes in the L-galactose pathway, and some of the genes encoding enzymes in the other three pathways, were identified in *A. latifolia* (Table 3), supporting that the predominant pathway of AsA biosynthesis in higher plants is the L-galactose pathway, which cooperates well with the other three alternative biosynthesis pathways [1,26].

In the present study, we quantified the global gene expression accompanying fruit development and ripening of *A. latifolia* and identified candidate genes associated with AsA biosynthesis and recycling (Figure 4 and Figure 5). Previous studies have demonstrated the correlation between *GMP* transcript level and AsA accumulation rate to some extent [23,27]. In this study, the *GMP* transcript level tended to be higher in the young fruits, and had a similar trend with AsA accumulation in early fruit developmental stages (Figure 6). *GMP* plays an important role in cell wall biosynthesis and protein glycosylation. Therefore, the two processes may also be involved in high *GMP* transcript levels in young fruits [27]. Transformation of plants with over-expression of the *GGP* gene in *Arabidopsis* resulted in a more than four-fold increase in AsA content [28]. As an early committed step of the L- galactose biosynthesis pathway, *GGP* contributes greatly to the rapid increase in AsA accumulation in several fruit species [23,29,30]. *GGP* has been considered the best regulatory control point for AsA biosynthesis [31]. In the present study, *GGP* showed relatively high expression levels in the early stages of fruit development (Figure 6), supporting the contribution of *GGP* to the AsA biosynthesis. In addition, the gene expression level of *GME* was also correlated with AsA content (Figure 6), emphasizing the central role and positive regulation of *GME* in AsA accumulation in immature fruit [23]. In addition to the three upstream enzymes, we observed a positive correlation between *GalDH*, *GPP*, *PGI*, *MIOX*, and *APX* and the cumulative AsA content (Figure 6), suggesting that the high expression of these enzymes resulted in high AsA accumulation in *A. latifolia*. Overall, the current study provided insights into the molecular mechanisms regulating AsA accumulation of *A. latifolia* fruit, which are expected to be useful for breeding cultivars with super-high AsA content in the future.

## 4. Materials and Methods

### 4.1. Plant Materials and Sampling

This study was conducted using 5-year-old (in 2019) fruit-bearing *A. latifolia* kiwifruit trees in an experimental block of Sichuan Provincial Academy of Natural Resource Sciences, Deyang, China (31°30′ N, 104°23′ E). Fruit samples were randomly collected from the fruiting branches (five per tree) of six different trees for AsA content determination and gene expression analysis. Fruit sampling included six representative stages of fruit development and ripening of *A. latifolia* corresponding to 30, 60, 90, 120, 150, and 170 days after flowering (DAF). The time when more than 75% of the flower’s petals had fallen was set as 0 DAF. Three biological replicates per stage were obtained from ten fruits per replicate. The samples were stored in a cold chamber and transported to the laboratory within 2 h. Upon arrival at the laboratory, samples were cut into slices, immediately frozen in liquid nitrogen, and kept at −80 °C until subsequent analysis.

### 4.2. Chemicals and Solvents

HPLC-grade authentic standards of AsA and oxalic acid were used (Beijing Solaribio Sciences & Technologies, Beijing, China). Ultrapure water with an electrical resistivity of 18.2 MΩ cm was prepared using a Milli-Q gradient water purification system (Millipore Corporation, Bedford, MA, USA) via a 0.22 μm filter. This purified water was used to prepare all solutions in this study.

### 4.3. Determination of AsA Using HPLC Coupled with UV Detection

AsA content was determined using HPLC coupled with UV detection, following the methods described by [32] with minor modifications. In brief, a portion of 2.0 g frozen samples was fully ground with 5.0 mL of 0.1% oxalic acid to a homogenous slurry until reaching a total volume of 25.0 mL. The extracts obtained were filtered through a 0.45 μm membrane before being considered ready for injection into the HPLC system. The AsA standard solution (1.0 mg/mL) was prepared by dissolving 25.0 mg AsA in 0.1% oxalic acid and diluted to 25.0 mL with the same solvent. HPLC analysis was performed using an Agilent 1260 HPLC instrument and a variable wavelength detector (Agilent, Santa Clara, CA, USA). The chromatographic separation was performed on a Zobax Stablebond Analytical SB-C_18_ column (250.0 × 4.6 mm, 5.0 μm). The mobile phase was a 0.1% oxalic acid solution. The flow rate of the mobile phase was kept constant at 1.0 mL/min and the injection volumes of the samples and standard were 10.0 μL for quantitative analysis. The UV absorbance of AsA was determined at 265.0 nm, and AsA content was quantified using a calibration curve.

### 4.4. RNA Extraction and Quality Evaluation

Total RNA was extracted using a PureLink RNA Mini Kit (Invitrogen Inc., Carlsbad, CA, USA) and purified using an on-column PureLink DNase treatment (Invitrogen Inc.) according to the manufacturer’s instructions. RNA purity was determined by A_260_ absorbance using a Nanodrop 2000 spectrophotometer (Thermo Fisher Scientific, Inc., CA, USA). RNA concentration was quantified using a Qubit 2.0 fluorometer (Invitrogen Inc.), and integrity was assessed using an RNA Nano 6000 Assay Kit on an Agilent Bioanalyzer 2100 system (Agilent Technologies, Santa Clara, CA, USA). The samples were sent to Biomarker Technologies Co. Ltd. (Beijing, China) for sequencing using PacBio and Illumina RNA-Seq technology.

### 4.5. Construction of Iso-seq cDNA Libraries and PacBio Sequencing

The mRNA was enriched using oligo-dT magnetic beads from 4.0 μg of total RNA and reverse transcribed into cDNA using the SMARTer^TM^ PCR cDNA Synthesis Kit (Clontech, now Takara, http://www.takarabio.com accessed on 10 February 2022). The size-selected cDNA library was constructed according to the BluePippin Size Selection System protocol, as described by PacBio (PN 100-092-800-03), and sequenced on the PacBio Sequel platform.

### 4.6. Reads Processing and Error Collection of PacBio Iso-seq Reads

Row data acquired from SMRT sequencing were processed using SMRTlink v5.0. The circular consensus sequence (CCS) reads were obtained from subread BAM files, and the full-length non-chimeric (FLNC) reads and non-full-length reads were determined by the simultaneous presence of the poly A tail signal and the 5′ and 3′ cDNA primers from reads of inserts (ROIs). The short reads were discarded. Subsequently, FLNC sequences were isoform-level clustered with iterative clustering for error correction (Quiver algorithm), and we generated one consensus isoform [33]. The non-full-length CCSs were polished using the Quiver algorithm. Finally, the isoform with a minimum Quiver accuracy of 0.99 was considered a high-quality isoform and used for subsequent analyses.

### 4.7. Gene Functional Annotation

All isoforms were subjected to functional annotation using multiple protein and nucleotide databases, including the National Center for Biotechnology Information (NCBI) non-redundant protein (Nr, cutoff E-value ≤ 1^e−5^) [34], gene ontology (GO, E-value ≤ 1^e−10^) [35], Kyoto Encyclopedia of Genes and Genomes (KEGG, E-value ≤ 1^e−3^) [36], clusters of orthologous groups (COG, E-value ≤ 1^e−3^) [37], eukaryotic orthologous groups (KOG, E-value ≤ 1^e−3^) [38], protein families (Pfam, E-value ≤ 0.01) [39], and a manually annotated and reviewed protein sequence database (Swiss-Prot, E-value ≤ 1^e−5^) [40].

### 4.8. Transcript Structure Analysis

Potential coding sequence (CDS) regions within the transcripts were predicted using TransDecoder (https://github.com/TransDecoder/TransDecoder/releases accessed on 13 May 2022). Simple sequence repeats (SSRs) within the transcriptome were identified using MISA (http://pgrc.ipk-gatersleben.de/misa/ accessed on 13 May 2022). LncRNAs were screened via the coding-non-coding-index (CNCI) with default parameters [41] and coding potential calculator (CPC) with the NCBI eukaryotes’ protein database (E-value < 1^e−10^) [42]. Each transcript was translated in three possible frames, and a Pfam Scan with default parameters of -E 0.001 --domE 0.001 was utilized to determine whether there exists a domain of a known protein family. Transcription factors (TFs) were predicted using iTAK software from putative protein sequences [43].

### 4.9. Illumina cDNA Library Construction and Second-Generation Sequencing for Transcriptome of Fruit Development and Ripening Stages

Eighteen cDNA libraries (six representative stages × three biological replicates) were constructed and used for second-generation high-throughput sequencing. RNA extraction and quality detection, cDNA synthesis, library preparation, high-throughput sequencing, identification of DEGs, functional categorization, and pathway analysis of DEGs followed our previously published protocol [9].

### 4.10. Validation of DEGs by Quantitative Reverse Transcription PCR (qRT-PCR)

Specific primers for qRT-PCR are presented in Table S1, which were designed using Primer Premier software (version 5.0; Premier Biosoft, Palo Alto, CA, USA). qRT-PCR and data normalization were performed as previously described by [9].

## 5. Conclusions

The lack of comprehensive genome sequence information limits the scope of investigation of the molecular genetic basis of *A. latifolia* with extremely high ascorbic acid content. In this study, we used PacBio SMRT sequencing technology to generate a high-quality, functionally annotated reference transcriptome for this kiwifruit species. This is the first *A. latifolia* FL transcriptome release covering fruit tissues extracted from six stages of fruit development and ripening, which will be crucial for both basic and applied research on biotechnology assays and genetic improvement in the future. Based on the transcriptome, pair-wise comparisons between different developmental stages were performed and differentially expressed genes (DEGs) were detected. The regulatory genes associated with the AsA biosynthesis and recycling pathways, and the lncRNAs involved in AsA-related gene expression regulation, were identified. In addition, the expression patterns of the genes involved in AsA biosynthesis and metabolism were further validated by qRT-PCR, which explains the high AsA content detected in *A. latifolia* fruit. Our study provides insights into the molecular mechanisms regulating AsA accumulation of *A. latifolia* fruit, which may facilitate the breeding of cultivars with super-high AsA content in the future.

## Figures and Tables

**Table 1:** Differentially expressed genes identified in the comparative Illumina transcriptome analysis.

Baseline Control	DAF60	DAF90	DAF120	DAF150	DAF170
DAF30	1871/1917	1628/1857	1931/2384	2373/2892	2632/3447
DAF60		1768/2147	1687/2288	2425/2911	2676/3441
DAF90			410/813	866/1513	1491/2412
DAF120				434/689	1167/1866
DAF150					643/1152

Note: The first column represents the baseline and the first line represents the treatment group. Red (first) and green (second) numbers represent significantly up- and down-regulated gene numbers, respectively. For example, ‘1871/1917’ in the second column and the second line means that a total of 1871 significantly up-regulated and 1917 down-regulated genes were identified in the comparison between DAF30_vs_DAF60 (the former was used as a baseline control, and the latter was the treatment group).

**Table 2:** Annotation summary of every DEG set among the six developmental stages of fruit development and ripening of *A. latifolia*.

DEG_set ^a^	Annotated ^b^	COG ^c^	GO ^d^	KEGG ^e^	KOG ^f^	Pfam ^g^	Swiss-Prot ^h^	eggNOG ^i^	Nr ^j^
DAF 30_vs_60	3718	1736	2940	1455	2053	3306	2983	3664	3709
DAF 30_vs_90	3405	1542	2704	1234	1760	3045	2756	3326	3381
DAF 30_vs_120	4232	1993	3435	1687	2207	3823	3474	4142	4209
DAF 30_vs_150	5176	2477	4187	2093	2771	4663	4232	5093	5156
DAF 30_vs_170	5991	2832	4841	2386	3305	5383	4807	5893	5964

^a^ DEG: differentially expressed gene. ^b^ Annotated: number of differentially expressed transcripts annotated. ^c^ COG: clusters of orthologous groups. ^d^ GO: gene ontology. ^e^ KEGG: Kyoto encyclopedia of genes and genomes. ^f^ KOG: eukaryotic orthologous groups. ^g^ Pfam: protein family. ^h^ Swiss-Prot: a manually annotated and reviewed protein sequences database. ^i^ eggNOG: evolutionary genealogy of genes, non-supervised orthologous groups. ^j^ Nr: non-redundant protein sequence database.

**Table 3:** Comparison of the number of gene families involved in the biosynthesis and recycling pathway of ascorbic acid in different kiwifruit genotypes.

Gene Name	Number of Genes				
	Hong Yang (*A. chinensis*) v2	Hong Yang (*A. chinensis*) v3	Red 5 (*A. chinensis*)	White (*A. eriantha*)	*A. latifolia*
PGI	5	4	4	6	6
PMI	2	5	4	5	2
PMM	10	8	7	8	14
GMP	3	1	3	3	8
GME	2	3	2	4	3
GGP	3	4	3	5	9
GPP	2	2	2	2	3
GalDH	1	1	1	1	1
GalLDH	1	1	1	1	1
MIOX	7	8	6	10	4
GalUR	5	5	3	6	2
AO	3	2	7	4	11
APX	12	4	11	17	17
DHAR	5	4	4	5	5
MDHAR	7	6	7	8	10

Note: The number of different genes in Hong Yang (v2/v3), Red 5, and White were determined by the search function of Kiwifruit Genome Database (http://kiwifruitgenome.org/ accessed on 6 March 2022).

## Data Availability

All data supporting the findings of this study are available within the paper and within its Supplementary Materials published online.

## Supplementary Materials

The following supporting information can be downloaded at: https://www.mdpi.com/article/10.3390/ijms23105808/s1.
