# Supplementary figures and images for: Dynamic Changes in Ascorbic Acid Content during Fruit Development and Ripening of Actinidia latifolia (an Ascorbate-Rich Fruit Crop) and the Associated Molecular Mechanisms
Source: Int J Mol Sci. 2022 May 22;23(10):5808. doi: 10.3390/ijms23105808 (PMC9146223; doi:10.3390/ijms23105808)

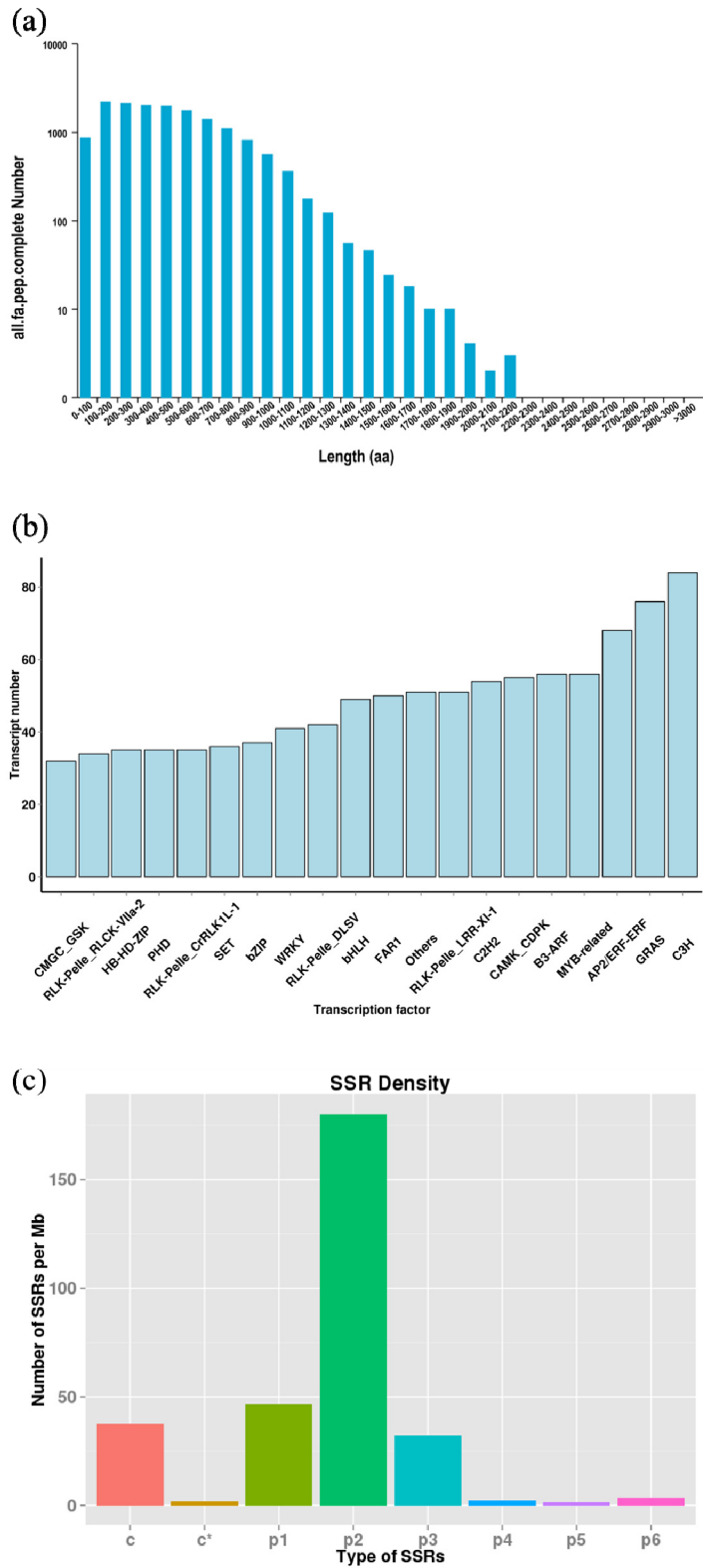



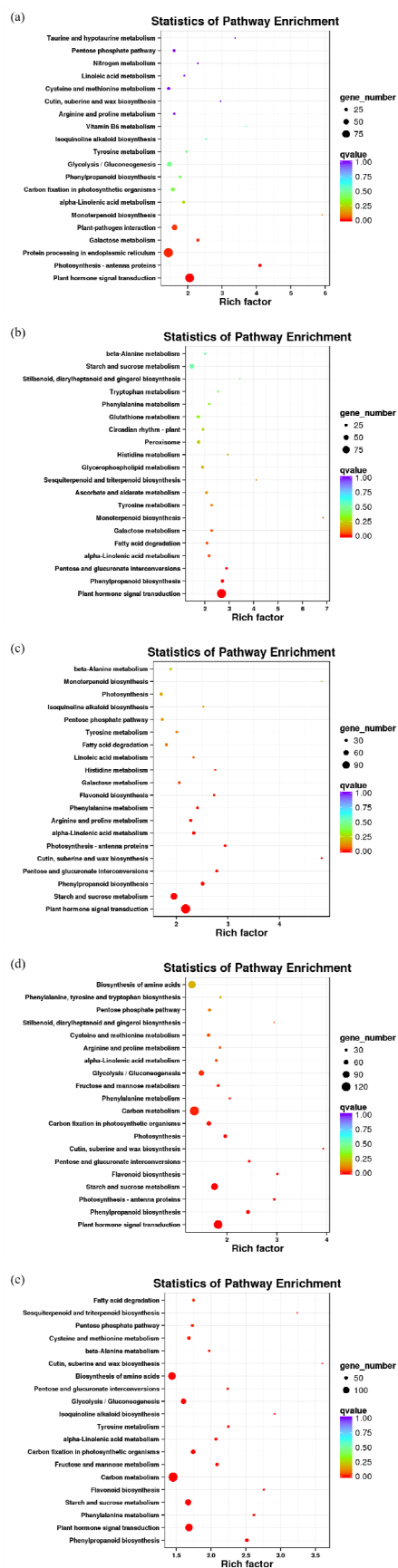

**Figure S3.** The top 20 enriched KEGG pathways of DEGs for CK (DAF30) *vs.* DAF60 (a), DAF90 (b), DAF120 (c), DAF150 (d), and DAD170 (e).

Supplement: Supplementary file 1 [file ijms-23-05808-s001.zip › Supplementary figures.pdf]
